# Brain activity associated with taste stimulation: A mechanism for neuroplastic change?

**DOI:** 10.1002/brb3.2928

**Published:** 2023-03-01

**Authors:** Angela M. Dietsch, Ross M. Westemeyer, Douglas H. Schultz

**Affiliations:** ^1^ Department of Special Education and Communication Disorders University of Nebraska‐Lincoln Lincoln Nebraska; ^2^ Center for Brain, Biology, and Behavior University of Nebraska‐Lincoln Lincoln Nebraska; ^3^ Department of Psychology University of Nebraska‐Lincoln Lincoln Nebraska

**Keywords:** functional magnetic resonance imaging (fMRI), sensorimotor, taste stimulation

## Abstract

**Purpose:**

Neuroplasticity may be enhanced by increasing brain activation and bloodflow in neural regions relevant to the target behavior. We administered precisely formulated and dosed taste stimuli to determine whether the associated brain activity patterns included areas that underlie swallowing control.

**Methods:**

Five taste stimuli (unflavored, sour, sweet‐sour, lemon, and orange suspensions) were administered in timing‐regulated and temperature‐controlled 3 mL doses via a customized pump/tubing system to 21 healthy adults during functional magnetic resonance imaging (fMRI). Whole‐brain analyses of fMRI data assessed main effects of taste stimulation as well as differential effects of taste profile.

**Results:**

Differences in brain activity associated with taste stimulation overall as well as specific stimulus type were observed in key taste and swallowing regions including the orbitofrontal cortex, insula, cingulate, and pre‐ and postcentral gyri. Overall, taste stimulation elicited increased activation in swallowing‐related brain regions compared to unflavored trials. Different patterns of blood oxygen level‐dependent (BOLD) signal were noted by taste profile. For most areas, sweet‐sour and sour trials elicited increases in BOLD compared to unflavored trials within that region, whereas lemon and orange trials yielded reductions in BOLD. This was despite identical concentrations of citric acid and sweetener in the lemon, orange, and sweet‐sour solutions.

**Conclusions:**

These results suggest that neural activity in swallowing‐relevant regions can be amplified with taste stimuli and may be differentially affected by specific properties within very similar taste profiles. These findings provide critical foundational information for interpreting disparities in previous studies of taste effects on brain activity and swallowing function, defining optimal stimuli to increase brain activity in swallowing‐relevant regions, and harnessing taste to enhance neuroplasticity and recovery for persons with swallowing disorders.

## Graphical abstract

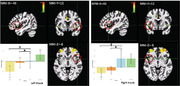

## 1 INTRODUCTION

For persons who develop neurogenic dysphagia, estimated to be 400,000–800,000 annually in the United States alone, the goal of restoring safe swallow function becomes a primary factor in resuming their former independence and quality of life (Panebianco et al., 2020). Rehabilitation of neurologically based swallowing disorders focuses on adaptation of the swallowing system. Historically, clinical intervention for dysphagia has mostly focused on peripheral adaptation, which might be achieved with strength‐based modalities such as tongue press against resistance, exhalation against resistance, tongue‐hold exercises, and head‐lifting exercises (Namasivayam‐MacDonald et al., 2022). There is reasonable evidence that such approaches increase the amplitude of muscle contractions, but it is less clear whether these enhancements translate to improved swallowing function (McKenna et al., 2017; Smaoui et al., 2020).

More centrally focused adaptation targets neuroplasticity, a reorganization of the neural underpinnings of the behavior of interest. It is well‐established that neuroplastic changes and motor relearning of complex behavior sequences such as swallowing rely on high‐quality, high‐repetition training that is salient to the targeted skill (Kleim & Jones, 2008; Schmidt & Wrisberg, 2004; Zimmerman et al., 2020). Repeatedly performing the behavior reinforces the underlying neural patterns and pathways, increasing the reliability and efficiency of the motor sequence. Additionally, the notion of metaplasticity suggests the synaptic state of neurons—and thus neuroplasticity—can be influenced by activity‐dependent stimulation that enhances the excitability of neurons (Abraham & Bear, 1996; Bienenstock et al., 1982). When such stimulation immediately precedes the salient high‐repetition training, it helps prime relevant clusters of neurons to fire together (Huang et al., 1992). These synaptic clusters become more cohesive, eventually influencing connectivity within and across regions to facilitate reorganization of the neural network underlying the target behavior. Thus, preswallow stimulation that successfully activates the neural areas involved in swallowing may give each practice swallow more rehabilitative potential.

A number of adjunctive therapies have been explored as potential modulators of cortical excitability in swallowing‐related areas. Direct means of modulating neural activity such as transcranial direct current stimulation (tDCS) and repetitive transcranial magnetic stimulation (rTMS) have shown promise for swallowing rehabilitation (Du et al., 2016; Papadopoulou et al., 2018; Pisegna et al., 2016). Somatosensory stimulation via vibration and electrical stimulation are linked to immediate increases in cerebral bloodflow and neural activity in sensory‐ and motor‐related cortical regions, suggesting metaplastic potential (Clark et al., 2009; Ludlow, 2010; Mulheren & Ludlow, 2017). However, these modalities are often unavailable in clinical settings and their long‐term effects on neuroplasticity have not yet been defined. More swallowing‐salient and readily available options for manipulating metaplasticity include sensory changes to the bolus itself, via temperature (Regan, 2020; Sciortino et al., 2003), carbonation and chemesthesis (Plonk et al., 2011; Todd et al., 2012), and taste (Babaei et al., 2010; Dietsch et al., 2019a; Logemann et al., 1995). In particular, taste stimulation has been linked to enhanced cortical activity in sensory and motor brain regions for swallowing in some studies (Abdul Wahab et al., 2010; Babaei et al., 2010; Dietsch et al., 2019b; Mulheren et al., 2016), and to more normalized swallowing kinematics in others (Dietsch et al., 2019a; Nagy et al., 2014; Pelletier & Dhanaraj, 2006). The latter is important in that neurostimulation approaches should also stabilize more normalized patterns of swallowing movement while avoiding any maladaptive ones.

Taste stimulation as an adjuvant to traditional motor‐based interventions is appealing because of its obvious salience to the act of swallowing, as well as significant overlap in cortical areas involved in the processing of taste sensation and the control of swallowing. Taste stimulation and sensitivity has been linked to activation in the primary gustatory cortex within the anterior insula, secondary gustatory cortex within the orbitofrontal cortex, oral and pharyngeal regions of the primary and secondary somatosensory cortices, and anterior cingulate cortex, among other regions (de Araujo et al., 2012; Eldeghaidy et al., 2011; van den Bosch et al., 2014). Swallowing is mediated by a complex neural network including cortical and subcortical structures such as the lateral inferior primary motor and somatosensory cortices (associated with oral/pharyngeal/laryngeal structures), premotor cortex, supplementary motor area, superior temporal gyrus, middle and inferior orbitofrontal gyri, insula, anterior cingulate cortex, thalamus, basal ganglia, and portions of the cerebellum (Kober, 2018; Kober et al., 2019; Malandraki et al., 2009; Martin et al., 2001, 2004). Additional brainstem structures such as the rostral nucleus tractus solitarius are also relevant for swallowing but are less amenable to neuroimaging in humans (Beckstead & Norgren, 1979). Thus, taste stimulation may effectively prime key areas of overlap including the anterior insula, orbitofrontal cortex, cingulate, and lateral somatosensory cortices, which then feed forward through the rest of the swallowing network to yield enhanced motor responses during subsequent swallows.

Despite this foundational support for the potential benefits of taste stimulation in dysphagia rehabilitation, clinical application has been stymied by the variability within the existing literature. First, many different taste stimuli have been trialed, with some more controlled than others in terms of composition, temperature, viscosity, and volume among other potentially relevant factors (Babaei et al., 2010; Dietsch et al., 2019a; Plonk et al., 2011; Sciortino et al., 2003). Another key limitation in translating taste‐related research to clinical practice is in the range of swallowing‐related outcomes reported, from ratings of airway compromise to detailed kinematic and morphometric data, electromyography, manometry, perceptual ratings, and various neuroimaging measures (Abdul Wahab et al., 2010; Mulheren et al., 2016, 2022; Regan, 2020; Sciortino et al., 2003). Furthermore, only a few studies assessed the same taste profiles across multiple outcome types that are relevant to neurorehabilitative potential. This variability makes it difficult to synthesize or extrapolate information across studies to arrive at clear clinical guidance.

In contrast, the specific taste solutions included in the current study were selected because they have previously been associated with immediate and beneficial changes to swallowing physiology in healthy and dysphagic participants as well as consistent perceptions regarding intensity and pleasantness (Dietsch et al., 2019a, 2014, 2019b; Nagy et al., 2014; Todd et al., 2012). While these desirable physiological adaptations are an important aspect of justifying a rehabilitation strategy, the next step is to determine if these same stimuli might contribute to longer‐term neuroplastic changes in the swallowing network.

To address this challenge, we designed an overarching project to systematically assess the neurological and behavioral effects of multiple precisely controlled taste stimulation in healthy individuals across ages and sexes. As previously reported, we identified that stimulus type and genetic taster status (GTS), an inherited relative sensitivity to taste properties linked to the TAS2R38 gene (Bartoshuk, 2000), interact such that specific tastants elicit different reactions in some taster profiles compared to others, in terms of brain activity and swallowing morphometrics (Dietsch et al., 2019b). However, GTS is not typically assessed in clinical settings. Therefore, it is important to consider the main effects of taste stimuli on neural activity (excluding GTS as a variable of non‐interest) as a potential means for enhancing neurorehabilitation of swallowing. Therefore, the current analysis sought to address two hypotheses:

H1: Regions associated with both taste processing and swallowing such as the anterior insula, orbitofrontal cortex, oral subregions of the somatosensory cortices, and anterior cingulate will exhibit different patterns of BOLD signal activity during stimulation with the four selected taste solutions compared to water trials.

H2: In these and other brain regions relevant to taste and swallowing, BOLD signal patterns will differ across the various taste stimuli.

## 2 METHODS

### 2.1 Participants

We recruited healthy women and men 19–49 years of age (Kennedy et al., 2010) who had no history of any condition that might have affected taste, swallowing, or underlying neurological status, including any orofacial injuries or surgeries (with the exception of routine wisdom tooth extractions). Furthermore, potential participants completed an MRI safety screening to rule out anyone with implanted metal or other conditions that would contraindicate MRI. Informed consent to the study protocol (as approved by the investigators’ Institutional Review Board #16267) was obtained from all participants. Twenty‐one persons were included in the final dataset (mean age 27.66 years; 11 women and 10 men; 6 supertasters, 6 midtasters, 9 nontasters as determined by PROP testing [Smutzer et al., 2013]; see Dietsch et al., 2019b for additional details). The distribution of genetic taster types is consistent with previously reported findings regarding the interaction of sex and GTS (Bartoshuk et al., 1994). All participants confirmed that they had not consumed any food/liquid nor tobacco products for at least 2 h prior to study participation, since satiety may influence neural responses to bolus stimuli (de Araujo et al., 2003). The body mass index of participants averaged 24.32, with participants spanning the underweight (*N* = 1), healthy weight (*N* = 11), and overweight (*N* = 9) categories as defined by the Centers for Disease Control and Prevention (CDC https://www.cdc.gov/obesity/basics/adult‐defining.html) in a distribution consistent with the general US adult population (Flegal et al., 2012).

### 2.2 Stimuli

For the fMRI dataset, five different liquids were prepared and administered. Taste stimuli included an intense sour (2.7% w/v citric acid), a sweet‐sour mixture (1.11% w/v citric acid + 8% w/v sucrose), and two variations on the sweet‐sour: an orange (1.11% w/v citric acid + 8% w/v sucrose + 1% v/v orange extract) and a lemon (1.11% w/v citric acid + 8% w/v sucrose + 1% v/v lemon extract) solution (Dietsch et al., 2019b; McBride & Johnson, 1987; Pelletier et al., 2004). All solutions were prepared in distilled water using food‐ or pharmaceutical‐grade components. Plain distilled water was used as a control condition. Although some studies suggest that water does have an appreciable “taste” (de Araujo et al., 2003; Rosen et al., 2010), the alternative control condition of no bolus presentation introduced additional differences in oral stimulation (such as somatosensory and temperature) compared to the taste trials which would have confounded results.

### 2.3 Data collection

The neuroimaging data for this study were collected using a Siemens 3T Magnetom Skyra with 32‐channel head coil over 4 runs of multiband fMRI (Gradient‐echo T2*‐weighted sparse echo‐planar imaging pulse sequence measuring blood oxygen level‐dependent (BOLD) signals with parameters: acquisition time per run = 6.93 min, GRAPPA, R = 2, voxel size 2.5 × 2.5 × 2.5 mm^3^, field of view = 210 mm, phase encoding = anterior‐posterior, TR = 1s, TE = 29.8 ms, flip angle = 60^o,^ bandwidth = 2055 matrix size = 84 × 84, multiband acceleration factor = 3). A high‐resolution anatomical image was also collected for precise alignment during image analysis (T1: acquisition time = 6:03 min, GRAPPA, R = 2, voxel size = 1 × 1 × 1 mm^3^, field of view = 256 mm, phase encoding = anterior‐posterior, TR = 2.2 s, TE = 3.37 ms, TI = 991 ms, flip angle = 7^o,^ bandwidth = 200 Hz, echo spacing = 7.9 ms).

**FIGURE 1:**
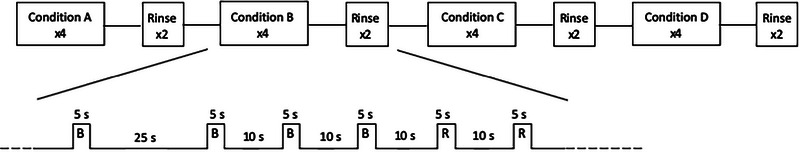
Each functional run included a sequence of four blocks (conditions A‐D, R = rinses between blocks). Within each block (expanded view with timeline), the participant received four trials of the same taste solution. Four counterbalanced functional runs were completed per participant, and multiband volumes were collected every one second throughout the run. Reprinted from Dietsch et al. (2019b). Genetic taster status as a mediator of neural activity and swallowing mechanics in healthy adults. *Frontiers in Neuroscience*, *13*(1328), p. 4. Reprinted with permission.

During acquisition of the task‐based fMRI, precisely timed 3 mL boluses of taste stimuli with intervening rinses were administered in a counterbalanced order via a customized delivery system. A PowerLab 16/35 (AD Instruments, Colorado Springs CO) and LabChart software (V. 8.1.13, AD Instruments, Colorado Springs CO) recorded TR pulses from the scanner, provided visual cues to the participants in the scanner, and controlled an array of modular pumps (Harvard Apparatus, Holliston MA). These pumps dispensed the liquids through a length of tubing (Skarda 1/8″ OD clear food‐grade urethane), the end of which was secured to the participant's lower face with medical tape such that the tip of the tubing was positioned in the anterior portion of the participant's oral cavity. Each condition block included four doses of the same taste solution (3 mL over 5 s each), with 15–30 s between onset of trials (see Figure 1). Each block was followed by a series of rinses via distilled water, administered through the same pump system. Because the shape of HDR specific to taste stimulation had not been previously reported in the literature (at the time of study design), the first interstimulus interval was a full 25 s to allow analysis of the complete HDR without interference from a subsequent stimulus. Analysis of these trials was used to identify the optimal parameters for all subsequent analyses of this dataset (Dietsch et al., 2019b). The other interstimulus intervals were shorter to balance the duration of each run with the number of trials administered.

As a separate part of the overarching study, participants completed generalized labeled magnitude scale (gLMS) ratings for intensity and hedonics immediately after presentation of each taste solution. As expected, intensity ratings were higher for supertasters than for nontasters for all taste profiles types, and all participants rated the intensity of each taste solution higher than for unflavored/water trials.

### 2.4 Analysis

MR preprocessing was completed using standard pipelines for preprocessing and whole‐brain analysis within and across subjects. This whole‐brain analysis evaluated whether the taste stimuli elicited different patterns of activation compared to the baseline (water) condition in any regions of the brain (H1). The steps, previously published in Dietsch et al. (2019b), were as follows:

Functional imaging reconstruction, processing, and analysis was conducted using Analysis of Functional Neuroimages (AFNI; Cox, 1996). Anatomical images were reconstructed and segmented using the standard Freesurfer processing pipeline (Desikan et al., 2006; Fischl et al., 2004). Anatomical images were then nonlinearly warped to MNI152_2009 template space using the AFNI program 3dQwarp, and the skull was removed. Echo‐planar images for each run were despiked (spikes in each voxel's time series are truncated), slice time corrected, aligned to the anatomical images and transformed to MNI space. Each volume was then registered to the volume with the minimum outlier fraction. Functional images were spatially smoothed using a 4 mm full‐width at half maximum Gaussian filter, and skull stripped. The time course of each voxel was scaled to a mean of 100. We then ran a general linear model using the six motion estimates from volume registration as regressors of no interest. Additionally, we used up to third‐order polynomials to model baseline and drift. Pairs of volumes where the Euclidean norm of the motion derivatives exceeded 0.4 were “scrubbed” and eliminated from further analysis. Finally, we modeled hemodynamic response functions using the “BLOCK” basis function at the onset time for each tastant as well as the rinse. The duration of the function was 6 s. Data from two participants (a female midtaster and a male nontaster) were excluded from further analysis due to technical issues with matching timelines of stimuli dispensation to image acquisition (pp. 4–5).

To determine whether there was a difference between BOLD activity during taste trials and the rinse (control) condition (H1), we calculated the mean beta weight for each voxel collapsed across the four taste stimuli for each participant. Next, we applied a cluster correction as follows:

A cluster‐based approach was used to correct for multiple comparisons (Forman et al., 1995). We estimated the spatial smoothness of the residuals for each participant using a Gaussian plus mono‐exponential function implemented with 3dFWHMx. The spatial autocorrelation function values were determined for each participant using the “‐acf” option, and the mean values across participants (0.761, 2.956, 11.06) were calculated (Cox et al., 2017). Ten thousand random maps with these smoothness parameters were generated and thresholded at a voxel‐wise *p* < .001. The largest surviving cluster from each of these simulations was recorded, and this distribution was used to estimate the probability of a false positive. Based on these estimations, we applied a cluster threshold to our data at a voxel‐wise *p*‐value of .001 and a minimum cluster size of ten contiguous voxels which resulted in a corrected two‐tailed alpha of *p* < .05. (Dietsch et al., 2019b, pp. 4, 5).

Then we conducted a paired *t*‐test between the mean taste betas and the betas for the rinse condition for each cluster using the AFNI program 3dttest++.

To address H2 regarding differences between taste solutions, we recalculated ANOVA within AFNI using taste profile as a within‐subjects factor in a method otherwise similar to what was described above. We also applied the same cluster‐based correction approach for multiple comparisons as described above.

Finally, beta weights for each surviving cluster associated with main effects of taste profile were extracted and subjected to repeated measures ANOVA via SPSS (IBM, Chicago IL). The associated post hoc tests assessed for significant differences between pairs of taste types with regard to BOLD response (H2).

## 3 RESULTS

**FIGURE 2:**
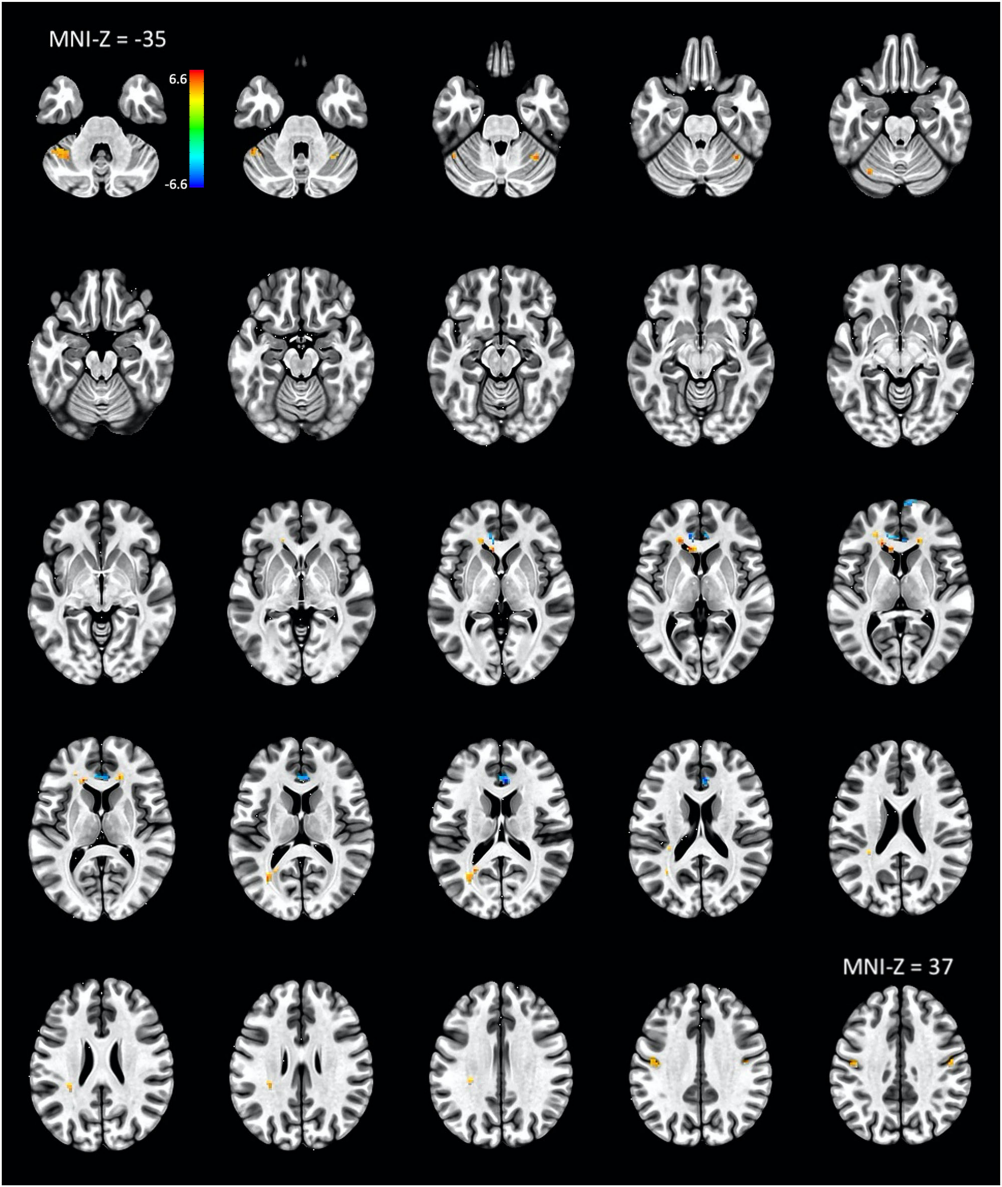
Superior view (5 mm slices) from MNI‐Z coordinates −35 mm through 37 mm show 11 clusters with statistically significant differences in activation during trials of taste stimulation (collapsed across sour, sweet‐sour, lemon, and orange) compared to water trials. The color bar reflects *t*‐values.

The analysis related to H1 revealed 11 areas exhibiting significant differences in activation during taste stimulation (collapsed across taste profiles) compared to the control (water) condition. Relevant statistics are shown in Table 1, and the locations of the clusters are reflected in Figure 2.

**TABLE 1:** Main effects of taste stimulation

	Coordinates (MNI152‐2009)				
Cluster	RL	AP	IS	Volume (mm^3^)	*t* Value
L anterior cingulate	−1	33	10	891	−4.581
L caudate	−15	30	5	875	4.753
L cerebellum (Crus 1)	−38	−51	−35	719	4.456
L postcentral gyrus	−44	−15	35	438	4.410
L middle temporal gyrus	−29	−63	15	360	4.547
R cerebellum (VI)	34	−55	−28	250	4.457
L anterior inferior insula	−30	−37	26	234	4.315
R postcentral gyrus	47	−14	36	188	4.349
R anterior cingulate	18	32	9	172	4.316
R superior frontal gyrus	12	70	7	172	−4.566
L cerebellum (VI)	−28	−68	−24	156	4.574

All taste profiles were concatenated and contrasted with the control (water) condition. Eleven clusters survived whole‐brain analysis. RL = right/left, AP = anterior/posterior, IS = inferior/superior.

The second analysis, examining differences in activation across taste stimuli (H2), yielded 18 clusters of statistical significance which are shown in Table 2.

**TABLE 2:** Differences in activation by taste stimulus

	Coordinates (MNI152‐2009)				
*Cluster*	RL	AP	IS	Volume (mm^3^)	*F*(1, 17)
R orbitofrontal gyrus	25	54	–4	5453	8.497
L orbitofrontal gyrus	–29	52	–4	2578	8.131
Middle anterior cingulate	0	5	38	1547	7.174
L superolateral postcentral gyrus	–37	–44	65	766	9.048
R supramarginal gyrus	63	–32	27	750	7.479
R superolateral postcentral gyrus	20	–32	63	594	8.614
L superior parietal lobule	–17	–60	68	328	8.500
R cuneus	13	–81	34	313	8.249
R precentral gyrus	32	–11	62	313	6.949
L cerebellum (VIII)	–22	–57	–50	203	7.032
L precuneus	–8	–50	61	203	7.922
R cerebellar vermis	2	–73	–39	188	7.260
L inferior frontal gyrus	–21	7	–23	188	8.323
L anterior inferior insular cortex	–45	12	–6	172	8.375
R anterior inferior insular cortex	46	12	–5	172	7.875
L precentral gyrus	–21	–16	67	172	7.828
R superior medial gyrus	12	63	9	156	7.259
L anterior cingulate/supplemental motor	–8	2	50	156	8.168

Eighteen clusters exhibited statistically significant differences in activation based on taste stimulus type during whole‐brain analysis and cluster‐based correction for multiple comparisons. RL = right/left, AP = anterior/posterior, IS = inferior/superior.

**FIGURE 3:**
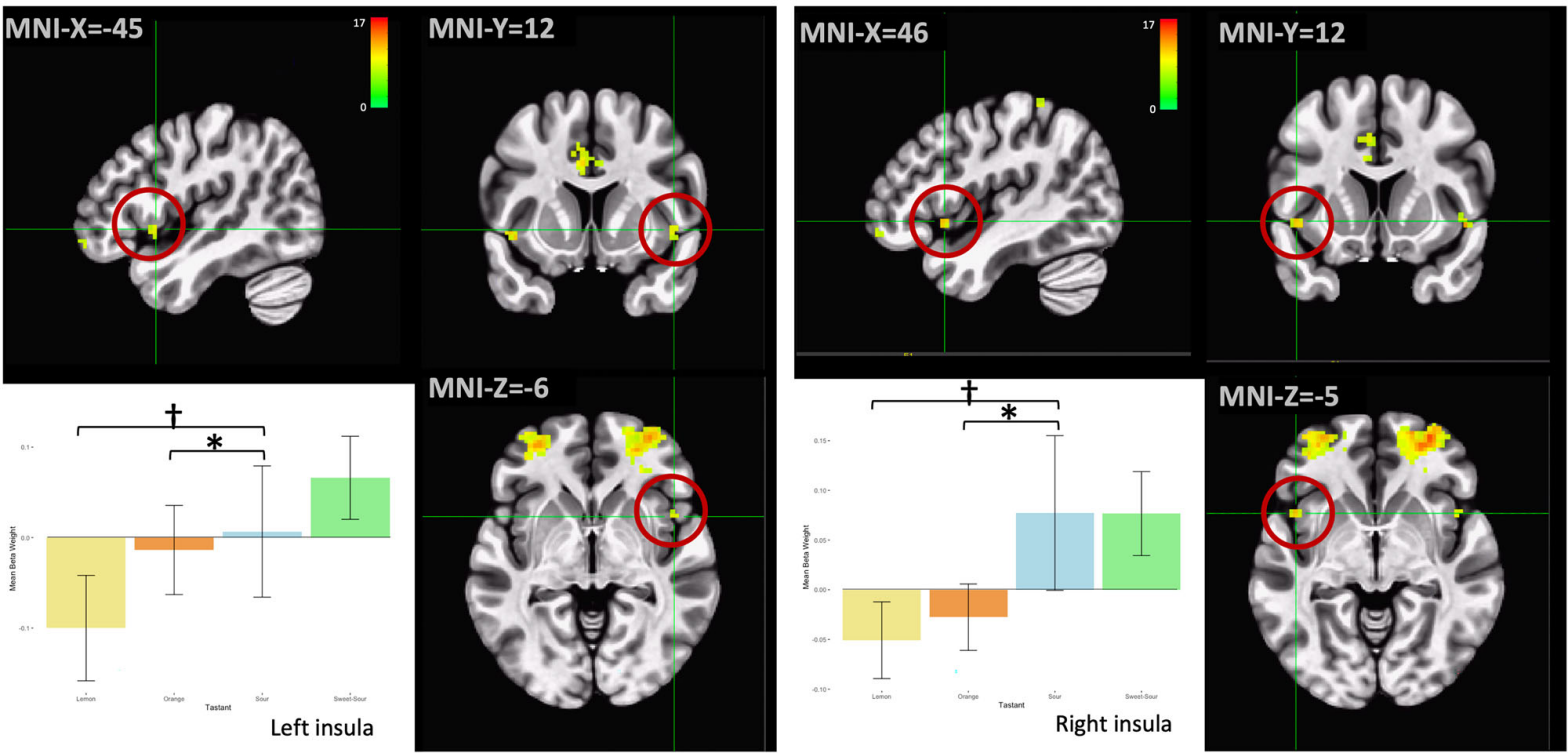
Regions in the bilateral anterior inferior insula, consistent with the primary gustatory cortex, were more active during sour and sweet‐sour trials than during the control condition and less active during lemon and orange trials. The magnitude of effect varied across taste stimuli and hemispheres. *p < .001, †*p* < .005, ‡*p* < .01 on post hoc Tukey's tests. Error bars represent ± 1 SE; the color bar reflects *F*‐values.

Clusters in the bilateral anterior inferior insula (Faillenot et al., 2017), which includes the primary gustatory cortex (de Araujo et al., 2012), exhibited depressed cortical activation with lemon and orange trials compared to water, whereas sour and sweet‐sour increased activation in these clusters. The magnitude of response for each taste solution differed between the right and left hemispheres (Figure 3). Despite this, post hoc Tukey's tests revealed statistically significant differences in the lemon/sour (*p* = .002 on each side) and orange/sour (*p* < .001 on each side) contrasts for both insular clusters.

**FIGURE 4:**
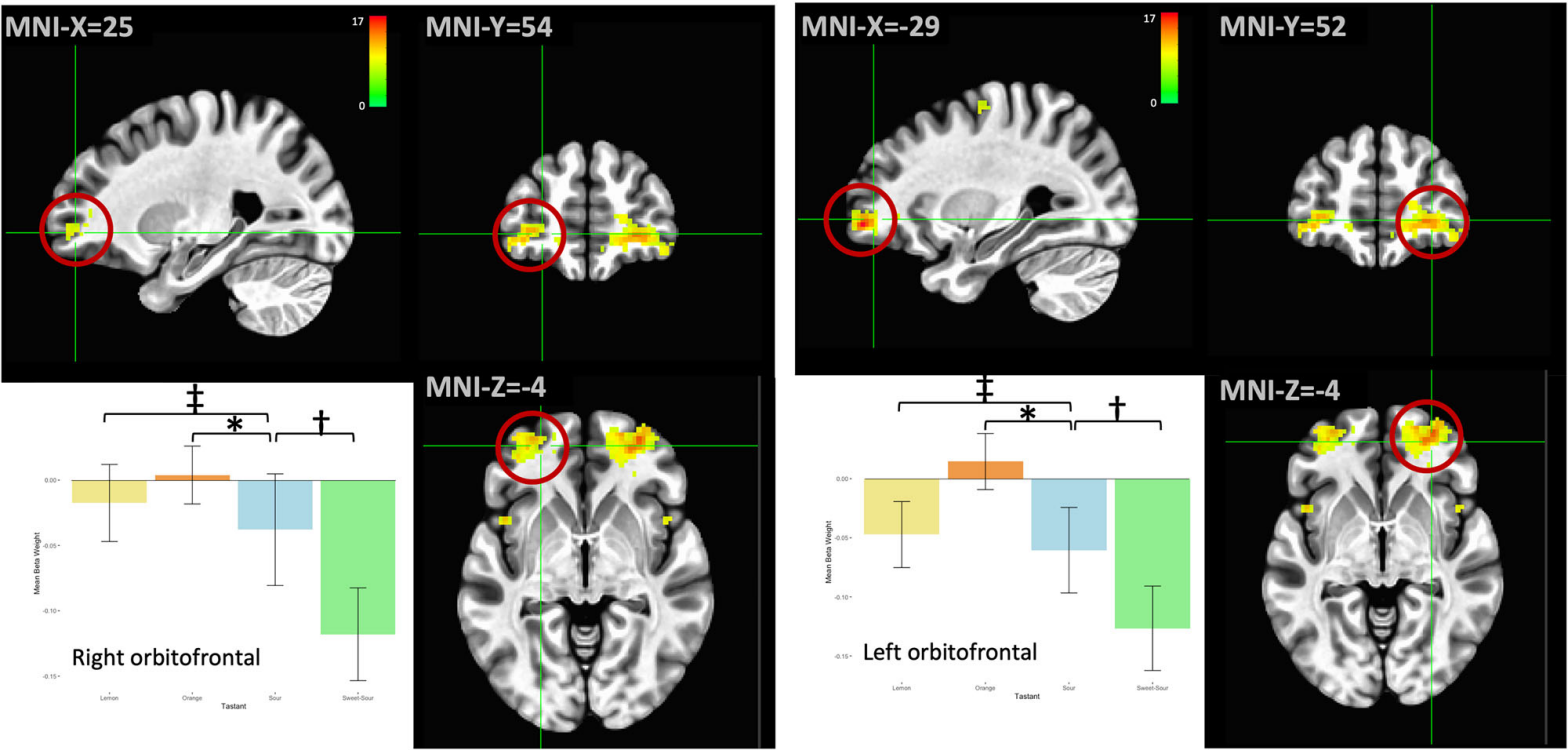
The secondary gustatory cortices within the bilateral orbitofrontal regions exhibited reduced activation (compared to control) during all trials except orange, which was associated with a mild increase in activation. In both hemispheres, response to sour was significantly different compared to all other taste profiles. *p < .001, †*p* < .005, ‡*p* < .01 on post hoc Tukey's tests. Error bars represent ± 1 SE; the color bar reflects *F*‐values.

Areas in the bilateral orbitofrontal cortices, where the secondary gustatory cortex is located (van den Bosch et al., 2014), were less active during most of the taste trials compared to the water condition. Only the orange trials were associated with increased activity in this region (Figure 4). Post hoc Tukey's results indicated statistically significant differences between sour and all three other taste profiles in the left (*p* = .006 for lemon/sour; *p* < .001 for orange/sour; *p* = .001 for sweet‐sour/sour) and right (*p* = .001 for lemon/sour; *p* < .001 for orange/sour and sweet‐sour/sour) orbitofrontal regions.

**FIGURE 5:**
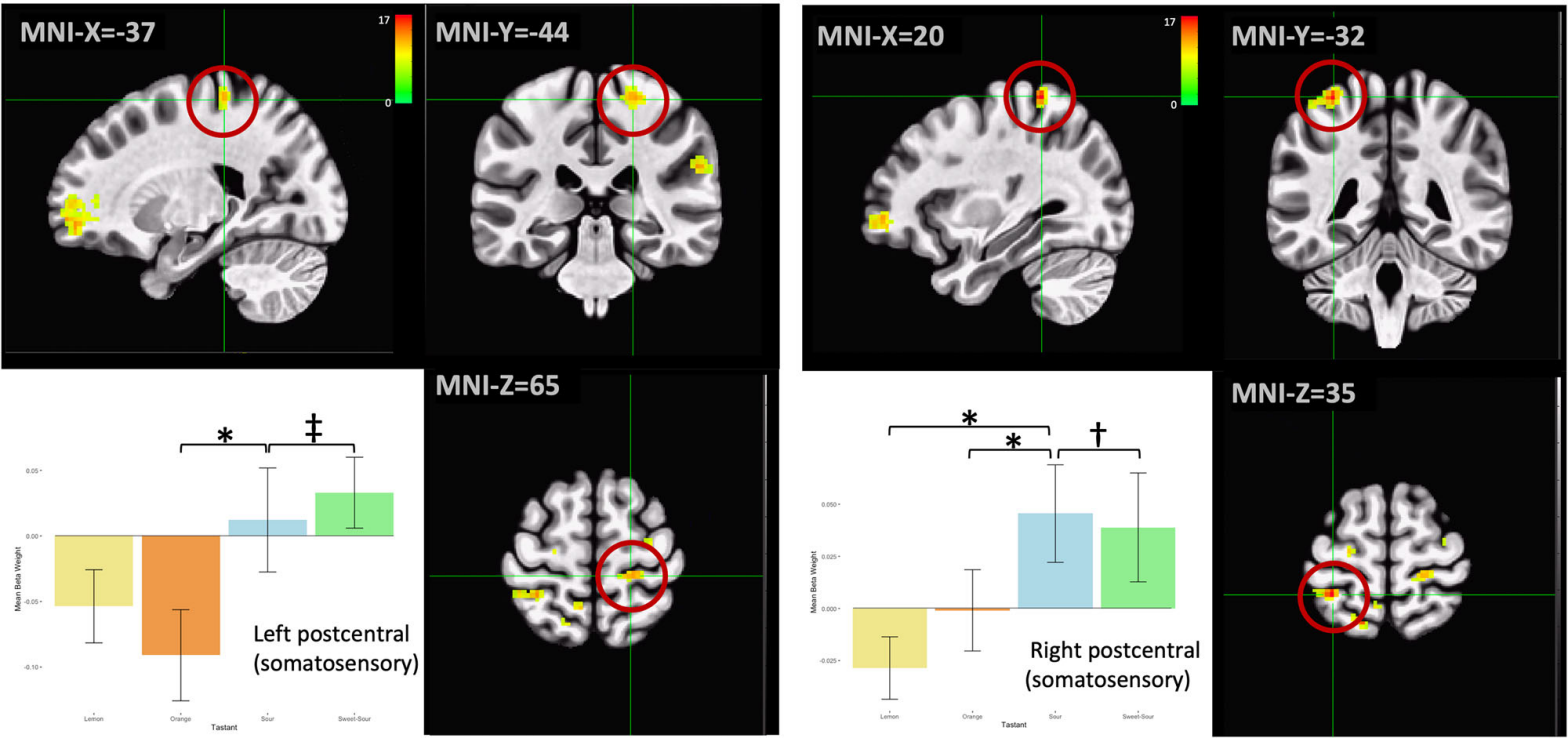
Regions within the superolateral somatosensory cortex were more active during trials of sour and sweet‐sour and less active during lemon and orange trials compared to the control condition. Significant differences in magnitude of response were observed across multiple pairs of taste stimuli. *p < .001, †*p* < .005, ‡*p* < .01 on post hoc Tukey's tests. Error bars represent ± 1 SE; the color bar reflects *F*‐values.

Compared to the unflavored condition, clusters within the subregions of the postcentral gyri's somatosensory cortices (S1) typically associated with oral sensation exhibited decreases in BOLD signal intensity during lemon and orange, whereas the sour and sweet‐sour were associated with increases in BOLD signal in S1 (Figure 5). Within the left S1, post hoc tests indicated statistically significant differences in the orange/sour (*p* < .001) and sweet‐sour/sour (*p* = .005) contrasts. In the right S1, the BOLD signal associated with sour trials was statistically significantly different between sour and each of the other taste profiles (*p* < .001 for lemon/sour and orange/sour; *p* = .002 for sweet‐sour/sour).

**FIGURE 6:**
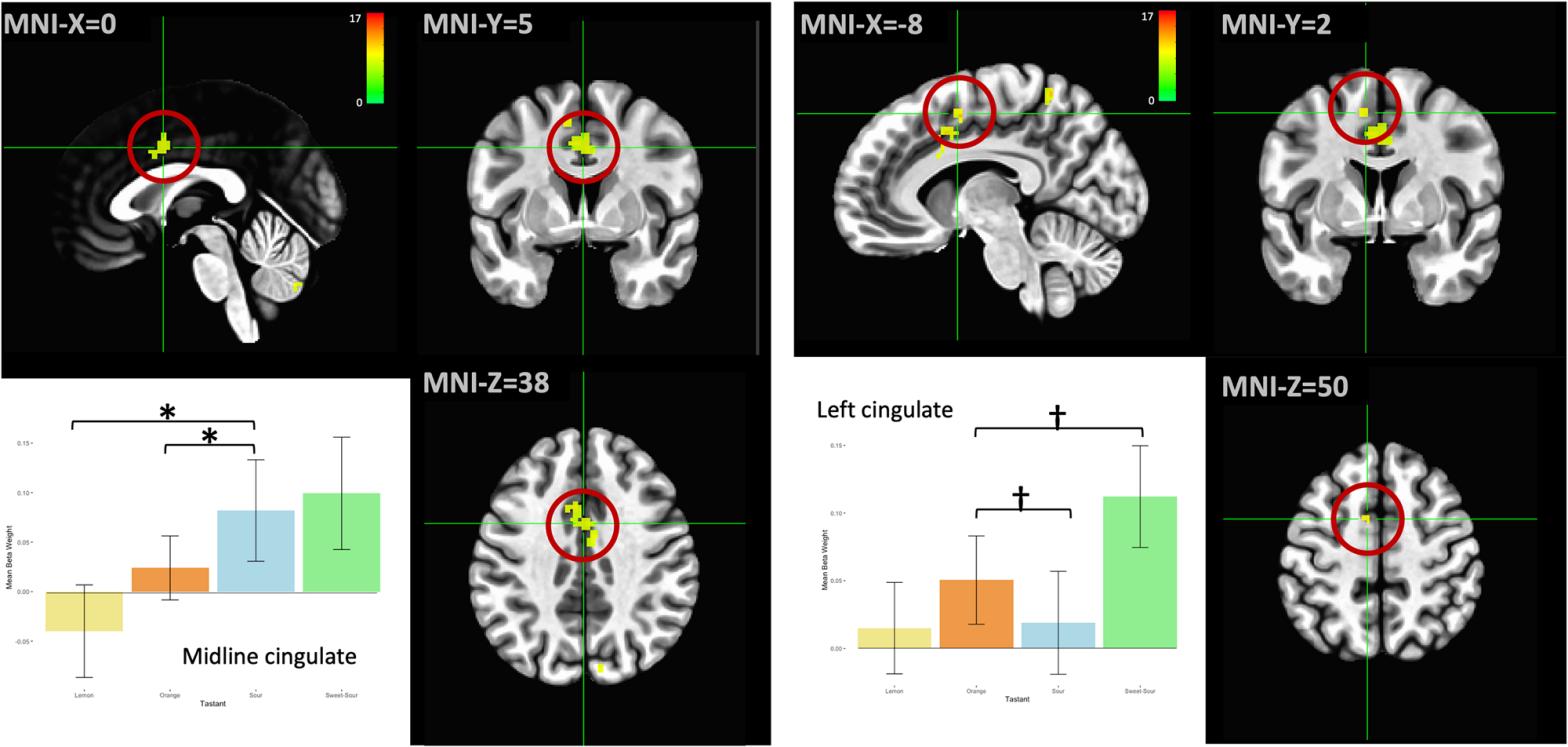
Regions within the middle anterior cingulate were more active during most taste trials compared to the control condition, with significant differences in magnitude of response across multiple pairs of taste stimuli. *p < .001, †*p* < .005, ‡*p* < .01 on post hoc Tukey's tests. Error bars represent ± 1 SE; the color bar reflects *F*‐values.

Two clusters within the middle anterior cingulate cortex near the supplemental motor area exhibited mostly increased activation during taste stimulation compared to the water condition (Figure 6). The lemon trial in the midline cluster was the only condition associated with decreased cingulate activity. The post hoc tests indicated that for the midline cluster, the lemon/sour and orange/sour contrasts were statistically significant (*p* < .001 for both pairs). In the left cingulate cluster, the orange/sour and orange/sweet‐sour contrasts were significant (*p* = .001 for both pairs).

Besides these four brain regions critical to sensory processing for taste, several other clusters showed statistically significant variations in BOLD responses according to taste stimuli. Clusters in the oral subregions of the bilateral primary motor cortices (MNI: 32, −11, 62 and −21, −16, 67) exhibited increased BOLD signal intensity with sour and sweet‐sour trials but not with orange and lemon trials. Clusters in the left and right cerebellum (MNI: −22, −57, −50 and 2, −73, −39, respectively) showed increased BOLD intensity with sour and sweet‐sour trials and decreased BOLD intensity with lemon trials. Other areas with smaller clusters of statistically significant differences in activation across taste type included the right inferior parietal cortex, the right cuneus, the right superior frontal gyrus, the left inferior frontal gyrus, and the left precuneus.

## 4 DISCUSSION

The purpose of this analysis was to characterize the effects of the specific taste stimuli assessed here on BOLD activity, with a long‐term objective of incorporating this taste stimulation into dysphagia therapy to enhance neurorehabilitation as well as swallowing physiology. The current results support the study hypotheses in that the taste stimuli were collectively associated with increased activation in brain regions that are well‐established as part of the swallowing network, and the taste profiles elicited different variations in BOLD signal within those regions.

The whole‐brain analysis revealed regions of activation that had significantly different patterns of activation during taste trials compared to the control condition (H1). These regions were largely consistent with expectations based on previous studies identifying key landmarks in the network associated with taste processing (Chikazoe et al., 2019; Small, 2010). Furthermore, there was significant overlap between these areas of taste‐associated BOLD change and the neural network underlying swallowing function. Taste‐dependent differences in BOLD signal (H2) were observed in seven of the eight regions predicted, with increases in BOLD intensity (compared to control) for some stimuli and decreases for others. Region‐by‐region consideration of these results complements and extends extant literature regarding their roles in the sensorimotor integration of taste as it relates to swallowing.

The anterior insula are the site of the primary gustatory cortices (de Araujo et al., 2012). They mediate the perception and recognition of taste stimuli as well as the integration of taste with nutritional status and olfactory, somatosensory, and visual inputs related to food. These activities contribute to taste perception and hunger. Additionally, the anterior insula are known to contribute to voluntary swallowing (Martin et al., 2001) and have long been implicated as important links between the primary and supplemental motor cortices and the nucleus tractus solitarius, two key regions for mediating oropharyngeal swallowing (Daniels & Foundas, 1997). Our results are consistent with previous work indicating that insular activation may vary by the magnitude of pleasantness and intensity of the taste stimuli (Cerf‐Ducastel et al., 2012). Although ratings of intensity and hedonics of the taste solutions were collected from participants during another phase of the overarching study, the sample size limits inclusion of such factors in the current analysis. Future work will incorporate such perceptual ratings as potential covariates to neural activations.

The bilateral orbitofrontal cortices (OFC) house the secondary gustatory cortices (van den Bosch et al., 2014). Activation in these areas has been positively correlated to the taster's ratings of pleasantness for a given taste stimulus, with the effect slightly stronger in the left side compared to the right (Kringelbach et al., 2003; Rolls et al., 1990). In this study, the orange liquid yielded slightly increased activity in the OFC compared to the control condition, whereas the other taste solutions were associated with less activity during trials. When considered in light of previous work, this could suggest that the orange profile was more appealing to the participants’ taste perception networks. Furthermore, the OFC is well‐established as part of the default mode network (DMN) which generally shows increased activity during rest and decreased activity during active task states, particularly for novel or high‐attention tasks (Bentley et al., 2016; Raichle, 2015; Shamloo & Helie, 2016). If that relationship is applied to the taste solutions in this study, orange and to a lesser extent lemon may have been more familiar or recognizable to participants whereas the intense sour and the sweet‐sour trials were perceived as comparatively more unusual or attention‐worthy. This is consistent with DMN activation observed during tasks with self‐referential associations or memories (Raichle, 2015). The OFC also has a role in swallowing control for water trials in healthy humans as well as automatic swallows in anesthetized sheep (Cheng et al., 2022). This has important implications for swallowing initiation, which is frequently compromised in neurologically based dysphagia and typically does not respond to strength‐based intervention approaches.

As part of the trigeminal pathway, S1 is involved in processing chemesthetic sensation from the oral cavity (Terrier et al., 2022). Chemesthesis includes the pucker‐inducing sensation associated with intensely sour stimuli. Compared to the other taste profiles in this study, the sour tastant had a much higher concentration of citric acid than the other stimuli and did not have additional flavor components to mask the sour. This more intense chemesthetic input may account for the statistically significantly amplified BOLD response to sour compared to all other solutions in the right S1 cluster. S1 is also foundational to the swallowing control network; sensory information about the bolus and the position of the oral structures is critical to coordinating the precise jaw and tongue movements required for mastication and deglutition (Cheng et al., 2022; Kober, 2018; Malandraki et al., 2009; Martin et al., 2004).

The two clusters in the middle anterior cingulate were near midline and on the left near Brodmann area 10. These regions are associated with sensory processing and interpretation, with specific roles in taste processing with activations sensitive to taste discrimination and pleasantness (Haase et al., 2011; Kringelbach et al., 2003). The cingulate receives input from the thalamus and has efferent projections to limbic areas as well as the supplemental motor area (Haase et al., 2011). The anterior cingulate is associated with voluntary bolus swallows but not automatic unstimulated swallows (Martin et al., 2001), which is particularly relevant for persons with dysphagia whose swallowing attempts and rehabilitation may elicit more cognitive attention and intention to the behavior than would occur for someone with an intact swallow during typical eating/drinking. In this study, taste‐associated changes within these cingulate clusters included increased BOLD activity in seven of eight cluster‐stimulus relationships. The varied directionality of statistically significant differences between stimulus types in each cluster may reflect that the somewhat different regions within the cingulate have different roles within the taste processing network.

In addition to the predicted regions of taste‐related change, there were clusters of significant taste‐related signal change in the bilateral primary motor cortices (M1) in the superolateral subregions typically associated with oral and pharyngeal/laryngeal structures. Here, sour and sweet‐sour were consistent in yielding increased BOLD signal intensity bilaterally. Inputs from M1 are critical to all aspects of bolus manipulation, mastication, and transit during oral stage swallowing, and appear to facilitate slight adjustments to pharyngeal swallowing morphometry (Ludlow, 2015). This supports the notion that taste stimulation could increase neuromodulation in brain regions that contribute to swallowing control. Though it is possible that some portion of the observed activation could reflect actual swallows that occurred intermittently during data collection, the differential response across multiple trials of the taste stimuli raises the likelihood that certain taste stimuli are contributing to increased M1 BOLD activity. Interpretation of these results are informed by studies using laminar MR (Huber et al., 2020; Persichetti et al., 2020) to describe differences in vascular space occupancy in M1 circuitry. They show that superficial layers of M1 (which are biased toward BOLD response because of the larger vessels) exhibit increased BOLD signal during imagined and executed motor tasks such as finger‐tapping, whereas deeper layers which are responsible for executing a motor signal are only activated during the executed tasks (Persichetti et al., 2020). Furthermore, the imagined task yielded sustained enhancement of the neural response during subsequent performed tasks. Extrapolating from these results, it is possible that taste stimulation could elicit a similar boost in neural responsivity that would have a beneficial effect on subsequent swallows; this could facilitate neural coupling and thus neuroplasticity in regions relevant to swallowing recovery.

There were also cerebellar regions that showed BOLD changes during taste stimulation compared to the control condition. These are consistent with findings of other authors that have described cerebellar activation patterns that appear to be involved in modulating oral and pharyngeal stage swallowing, particularly in response to taste stimuli (Mottolese et al., 2013; Small et al., 2003). These may represent important connections between the brainstem and cortical areas involved in swallowing (Rajappa & Malandraki, 2016). Together with the current findings, this suggests that even difficult‐to‐image brainstem areas relevant to swallowing control are affected by taste stimulation and more specifically, taste profile. From a metaplasticity standpoint, the opportunity for taste solutions that are already known to enhance swallowing physiology to also engage connected regions in the brainstem and cortical areas during swallowing could be an important contributor to functional recovery of swallowing.

With regard to the second hypothesis, taste‐dependent differences in BOLD were observed in all of the regions predicted. Within the bilateral insula, orbitofrontal cortex, somatosensory cortices, and left cingulate, at least some taste profiles were associated with decreases in BOLD activation whereas others had increased signal or no significant change. The midline cingulate was the only exception, with all four taste stimuli eliciting increased activation. Sour and sweet‐sour generally shifted in the same direction (though not necessarily the same magnitude) within a cluster, meaning that if BOLD increased with sour, it also increased with sweet‐sour but not lemon or orange. The sour tastant had a higher concentration of citric acid than the other three solutions, but the sweet‐sour was identical to the lemon and orange in all ways except the addition of 1% v/v lemon or orange extract. It is possible that the flavor extracts provided additional sensory input via gustatory, chemesthetic, or even olfactory channels, or that the perceptual representation of the stimulation profiles elicited a different associated response. Stronger responses to sweet‐sour than lemon and to lemon than orange could suggest that whatever olfactory sensation or associations with recognizable flavor profiles exist, they do not consistently increase neural engagement over and above the effect of taste in neural regions relevant to both by taste processing and swallowing control. Future studies with expanded sample sizes could help illuminate how multisensory stimulation interacts with sensory perception and even GTS to optimize stimulus selection for individuals seeking dysphagia rehabilitation.

Like all studies, ours has certain limitations. The sample is diverse across age, sex, and GTS but its size prevents meaningful analysis of the interaction of these factors on taste effects. The current results reflect the effects of taste on neurotypical brains; those with neuropathology may not respond in the same ways to taste stimulation or may have variations in the swallowing control network. Also, the 3T MRI used here cannot discern between superficial and deep activations in M1, so the laminar effects of taste on neural activation cannot be fully appreciated (Huber et al., 2020; Persichetti et al., 2020).

At least three critical next steps in this line of inquiry will inform the clinical application of our current findings. Analysis of functional and structural connectivity among regions of taste‐ and swallowing‐related activity will help inform how taste stimulation might contribute to the current results. For example, the orbitofrontal cortices have afferent connections from the primary gustatory, cingulate, and somatosensory cortices (Rolls, 2000, 2004), and efferent projections to the limbic system (Rolls et al., 1990) so communication across these network connections could contribute to BOLD signal changes observed in these regions. It may also help identify how other areas of taste‐associated BOLD signal change identified in the current study, such as the right inferior parietal and left inferior frontal lobes, contribute to interpretation of sensory information and sensorimotor integration for swallowing. Additionally, these results must be considered in conjunction with the interaction of taste stimulus type and genetic taster status (Dietsch et al., 2019b), since together these may be a relevant consideration for optimizing future interventions. Finally, we will examine strategies for safely delivering taste stimulation in a dysphagic population, including the use of dissolvable taste strips that are perceptually and chemically similar to the liquid taste stimuli tested here. All of these will help delineate how stimulation using specific taste profiles can enhance neuroplasticity for swallowing rehabilitation.

To summarize, we found that there were significant differences in neural activity in response to taste and based on taste in healthy adults. In general, lemon and orange solutions yielded reduced BOLD signal in key regions that overlap for taste and swallowing functions, whereas the identical‐intensity sweet‐sour as well as sour trials were associated with increased BOLD signal in those regions. These findings align with key factors that are thought to facilitate neurorehabilitation of swallowing, including the neuromodulatory power of salient sensory inputs (vibration, electrical stimulation, and now perhaps taste) that are associated with improved functional behavior and increased neural activation in relevant brain regions (Martin, 2009). More simply, the increased neural activations in brain regions that are associated with the taste stimuli tested here may have potential to enhance the neuroplastic benefits of dysphagia intervention. Although these preliminary results need to be confirmed in other cohorts, there are several potential implications. First, in order to maximize metaplasticity benefits, we may need to consider taste‐ and patient‐specific characteristics that could make one taste profile more beneficial than another for a particular person. Second, it is possible that the differences identified here could help explain some of the disparities in previous neuroimaging studies regarding the effects of taste stimulation. Further systematic exploration of the relationship between taste stimulation, neural activity, and swallowing holds promise as a means to transform dysphagia rehabilitation.

## CONFLICT OF INTEREST STATEMENT

The authors have no known conflicts of interest to disclose.

### ETHICS STATEMENT

This research was approved by the Institutional Review Board at The University of Nebraska‐Lincoln. All enrolled participants provided informed consent for this study. Portions of content in the methods section are reproduced from Dietsch et al. (2019b) under Creative Commons CC‐BY license and are attributed as required.

### PEER REVIEW

The peer review history for this article is available at https://publons.com/publon/10.1002/brb3.2928.

## Data Availability

The data that support the findings of this study are available from the corresponding author upon reasonable request.
